# Therapeutic efficacy of quercetin in experimental pulmonary fibrosis: A meta-analysis

**DOI:** 10.3892/etm.2025.13054

**Published:** 2025-12-22

**Authors:** Lanhua Cai, Long Chen, Chunmei Lin

**Affiliations:** 1 Department of Traditional Chinese Medicine, Guangzhou Red Cross Hospital of Jinan University, Jinan University, Guangzhou, Guangdong 510000, P.R. China; 2 Department of Prenatal Diagnosis Center, Maoming People's Hospital, Maoming, Guangdong 525000, P.R. China; 3 Department of Traditional Chinese Medicine, The Affiliated Guangdong Second Provincial General Hospital of Jinan University, Guangzhou, Guangdong 510000, P.R. China

**Keywords:** quercetin, pulmonary fibrosis, therapeutic efficacy, animal models, meta-analysis

## Abstract

Pulmonary fibrosis (PF) is a fatal chronic disease characterized by progressive interstitial scarring, alveolar destruction and a persistent decline in lung function. The present study evaluated the therapeutic effects of quercetin, a natural flavonoid with potent antioxidant and anti-inflammatory properties, in animal models of PF (PROSPERO registration no. INPLASY202530122). A comprehensive literature search was performed across PubMed, Web of Science, Embase, Cochrane Library and Ovid from inception to January 2025, identifying 25 eligible preclinical studies. Data were extracted and analyzed for outcomes in three main categories: Fibrotic changes, inflammatory responses and oxidative stress parameters. Random-effects meta-analysis was employed, followed by meta-regression analysis to examine study heterogeneity. The analysis indicated that quercetin administration was associated with improvements in fibrotic indicators, showing reduced collagen deposition and improved histopathological scores. Anti-inflammatory effects were observed through modulation of cytokine levels and inflammatory cell infiltration. Additionally, quercetin demonstrated effects on oxidative stress markers, showing enhanced antioxidant capacity and reduced oxidative damage. Meta-regression analysis identified a number of moderating factors, including dosage, treatment duration, animal model selection and induction method, which contributed to heterogeneity across studies. The findings suggest that quercetin may provide beneficial effects in experimental PF models through multiple pathways. However, notable heterogeneity was observed among included studies, indicating the need for cautious interpretation of results. Further investigation with standardized protocols is recommended to validate these preliminary findings.

## Introduction

Pulmonary fibrosis (PF) is a chronic, fatal disease characterized by progressive lung tissue scarring and functional decline, ultimately leading to impaired gas exchange. The core pathological mechanisms involve aberrant fibroblast activation, excessive extracellular matrix deposition, persistent oxidative stress and chronic inflammation (1,2). Idiopathic PF, the most common form of PF, has a median survival of only 2.5-3.5 years and to the best of our knowledge, there is no curative treatment at present (3). Present clinical therapies, such as pirfenidone and nintedanib, can only slow disease progression but are associated with side effects, including gastrointestinal disturbances and hepatic toxicity (4). Given these limitations, naturally derived compounds such as quercetin have garnered notable translational interest due to their multi-target mechanisms and favorable safety profiles. Therefore, exploring naturally derived compounds with favorable safety profiles to intervene in the fibrotic process has become a promising area of research.

Quercetin (3,5,7,3',4'-pentahydroxyflavone) is a natural flavonoid compound found in plants such as apples, onions and tea and in Traditional Chinese Medicine, is known for its multifaceted biological activities (5,6). In recent years, an increasing number of studies have demonstrated the potential therapeutic effects of quercetin in PF. Research has shown that quercetin can inhibit the onset and progression of PF through numerous mechanisms, such as suppressing the production of inflammatory cytokines, promoting apoptosis and inhibiting the expression and activity of TGF-β1 (7-9). Furthermore, quercetin exhibits a good safety profile, is low-cost and readily accessible (10), making it a notable area of research interest. Existing studies (11,12) primarily focus on animal models and *in vitro* mechanisms, while clinical translational evidence remains insufficient. In addition, while *in vitro* and animal studies provide valuable evidence, the translational potential of quercetin may be affected by the fragmented nature of preclinical data and methodological heterogeneity.

To address the aforementioned issues, the present meta-analysis consolidates preclinical evidence to quantitatively assess the efficacy of quercetin in improving outcomes related to PF. By accumulating data on fibrotic markers, inflammatory cytokines and oxidative stress parameters, the present review systematically evaluates the therapeutic effects of quercetin in PF. The findings aim to provide a theoretical basis for clinical trials exploring quercetin as an adjunctive treatment for PF, advancing its experimental potential toward clinical efficacy.

## Materials and methods

### Literature search strategy

A systematic search was performed across PubMed (https://pubmed.ncbi.nlm.nih.gov/), Web of Science (https://www.webofscience.com/), Embase (https://www.embase.com/), Ovid (https://ovidsp.ovid.com/) and Cochrane Library (https://www.cochranelibrary.com/) databases between inception and January 1, 2025. Following the population, intervention, comparison, outcomes and study (PICOS) framework (13), the search strategy was designed using the keywords ‘quercetin’ and ‘pulmonary fibrosis’, including both subject headings and free terms. The detailed search strategy is provided in Tables SI, SII, SIII, SIV and SV. The search approach included keywords, full-text searches and Medical Subject Headings, with no restrictions on publication type, sample size, study design or methods of exposure or outcome measurement. In addition, gray literature was manually searched using Google Scholar (https://scholar.google.com/). The present study is a secondary analysis and did not require ethical approval for animal or human experiments.

### Inclusion and exclusion criteria

Inclusion criteria were established based on the PICOS framework and were as follows: i) Population [animal models of PF (rodents)]; ii) intervention (monotherapy with quercetin or its derivatives); iii) comparator (placebo or blank control); iv) outcome; and v) study design (randomized or non-randomized controlled trials with full text available).

Several outcome measures were considered suitable. The outcome measures assessed were: i) Basic characteristics [body weight and lung index (lung index=lung weight (mg)/body weight (g)]; ii) fibrosis markers [hydroxyproline content, Ashcroft score (14), α-smooth muscle actin (α-SMA) and collagen I (Col I)]; iii) inflammatory cytokines (TNF-α, IL-6, IL-1β, TGF-β1, total cell count, leukocyte count, neutrophils, lymphocytes, eosinophils and macrophages); and iv) oxidative stress indictors [superoxide dismutase (SOD) activity, malondialdehyde (MDA) levels, glutathione (GSH), catalase (CAT), nitric oxide (NO) and thiobarbituric acid-reactive substances (TBARS)].

Exclusion criteria for article screening were as follows: i) Exclusively *in vitro* studies, clinical trials, reviews or conference abstracts; and ii) incomplete data (for example, charts with unannotated values), duplicate publications or studies where the full text was unavailable.

### Literature screening and data extraction

For screening, two independent researchers conducted separate literature searches using the predefined search strategy. The search results were imported into NoteExpress software (version 4.0.0.9855; Beijing Aegean Software Co., Ltd.) and checked for duplicates. Next, the titles and abstracts were initially screened according to the inclusion and exclusion criteria, after which the full texts of the selected studies were read for further screening. The basic information of the studies that were ultimately included was extracted. Any disagreements were resolved through discussion or consultation with a third-party expert to reach a consensus. Data extraction followed the guidelines outlined in the Preferred Reporting Items for Systematic Reviews and Meta-Analyses (15) statement. The following data were extracted from the studies: i) Study characteristics, authors, publication year, species/strain, sex, age, sample size and modeling methods; ii) intervention details, quercetin dosage, administration route and intervention duration; iii) outcome measures, mean values, SD and sample size (n) for both experimental and control groups; and iv) methodological quality, information on randomization, blinding and allocation concealment, among others. If different dosages were used in the studies, the highest dosage results were extracted. When results were measured at multiple time points, data from the longest duration were recorded. If the data were presented solely in graphical form, the authors were contacted to obtain the raw data. If no response was received, numerical values were extracted using Engauge Digitizer software (version 12.1; Engauge Open Source Developers). If different values were obtained by the two researchers, the mean of the value was calculated to produce a single estimate for analysis, thereby reducing measurement error.

### Quality assessment/bias risk analysis

The risk of bias in animal studies was assessed using the SYRCLE risk of bias tool (16), which includes 10 categories: i) Sequence generation; ii) baseline characteristics; iii) allocation concealment; iv) random housing; v) blinding implementation; vi) random outcome assessment; vii) blinded outcome assessment; viii) incomplete outcome data; ix) selective outcome reporting; and x) other sources of bias. Each category was rated as high, unclear or low risk of bias. Quality assessment was performed by three researchers and any discrepancies in the ratings were resolved through discussion.

### Statistical analysis

Data analysis was performed using RevMan (version 5.4; The Cochrane Collaboration) and Stata (version 17.0; StataCorp LP) software. Continuous variables are expressed as standardized mean differences (SMDs) with 95% CI. Heterogeneity was assessed using the I^2^ statistic (with I^2^ >50% indicating significant heterogeneity). A random-effects model was applied when I^2^ >50%, while a fixed-effects model was used when I^2^ ≤50%. To explore potential sources of heterogeneity, an exploratory meta-regression analysis was performed on all available data, incorporating study characteristics as covariates. Publication bias was evaluated both visually using funnel plots and statistically using Egger's linear regression test and Begg's rank correlation test. P<0.05 was considered to indicate a statistically significant difference.

## Results

### Search results

**Figure 1:**
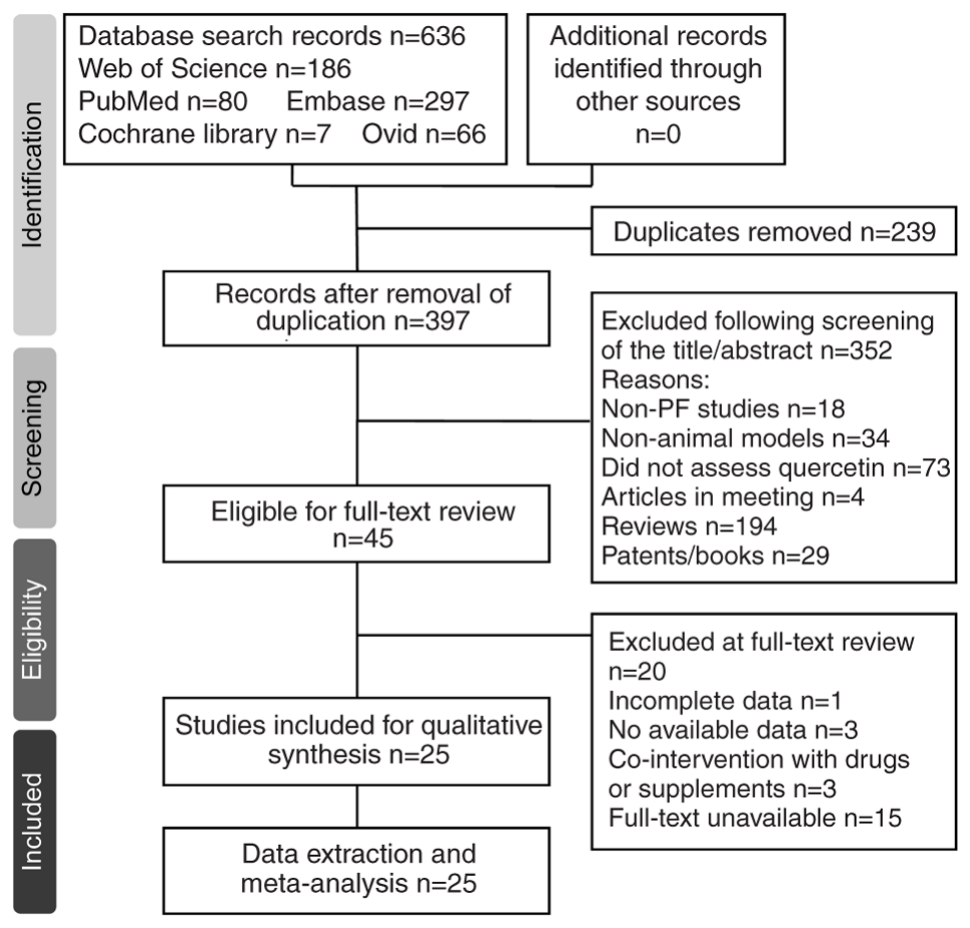
Flow diagram of the screening and selection process. PF, pulmonary fibrosis.

Literature searches yielded a total of 636 articles (PubMed, 80; Web of Science, 186; Embase, 297; Cochrane Library, 7; Ovid, 66). After removing 23 duplicated articles, 613 articles were screened based on titles and abstracts, resulting in the exclusion of 352 studies that did not meet the inclusion criteria. Full-text evaluation was performed on 45 articles, leading to the exclusion of 20 studies due to irrelevance or incomplete data. Manual searching on Google Scholar did not reveal any additional qualifying studies. Ultimately, 25 studies (17-41) were included in the present meta-analysis (Fig. 1). The basic characteristics of the included studies are shown in Table I.

### Basic characteristics of included studies

A total of 25 studies were included in the analysis, performed across 11 countries. Among these, 12 studies were from China, 2 each from India, Iran and Turkey and 1 each from the United States, Brazil, Egypt, Germany, Italy, South Korea and Nigeria. All studies were preclinical controlled trials utilizing rat models. Of these, 24 studies used quercetin as the sole intervention, while one study employed a derivative of quercetin. The experimental animals included male rats (60%), female rats (24%), mixed sex (8%) and those with unspecified sex (8%). The age of the animals varied notably, ranging from 6 weeks to >12 months, with 10 studies not reporting the age. The primary outcome measures included body weight (5 studies), lung index (4 studies), fibrosis markers (hydroxyproline content in 9 studies, α-SMA in 6 studies and COL I in 4 studies), histopathological scores (Ashcroft score in 7 studies), inflammatory markers (TNF-α in 11 studies, IL-1β in 6 studies and IL-6 in 5 studies), inflammatory cell counts (total cell count in 7 studies, macrophage count in 7 studies, neutrophil count in 5 studies, lymphocyte count in 4 studies, eosinophil count in 3 studies and white blood cell count in 3 studies) and oxidative stress markers (MDA levels in 7 studies, GSH levels in 6 studies, SOD and CAT enzyme activities in 4 studies each and TBARS in 3 studies).

### Quality assessment of the studies

**Figure 2:**
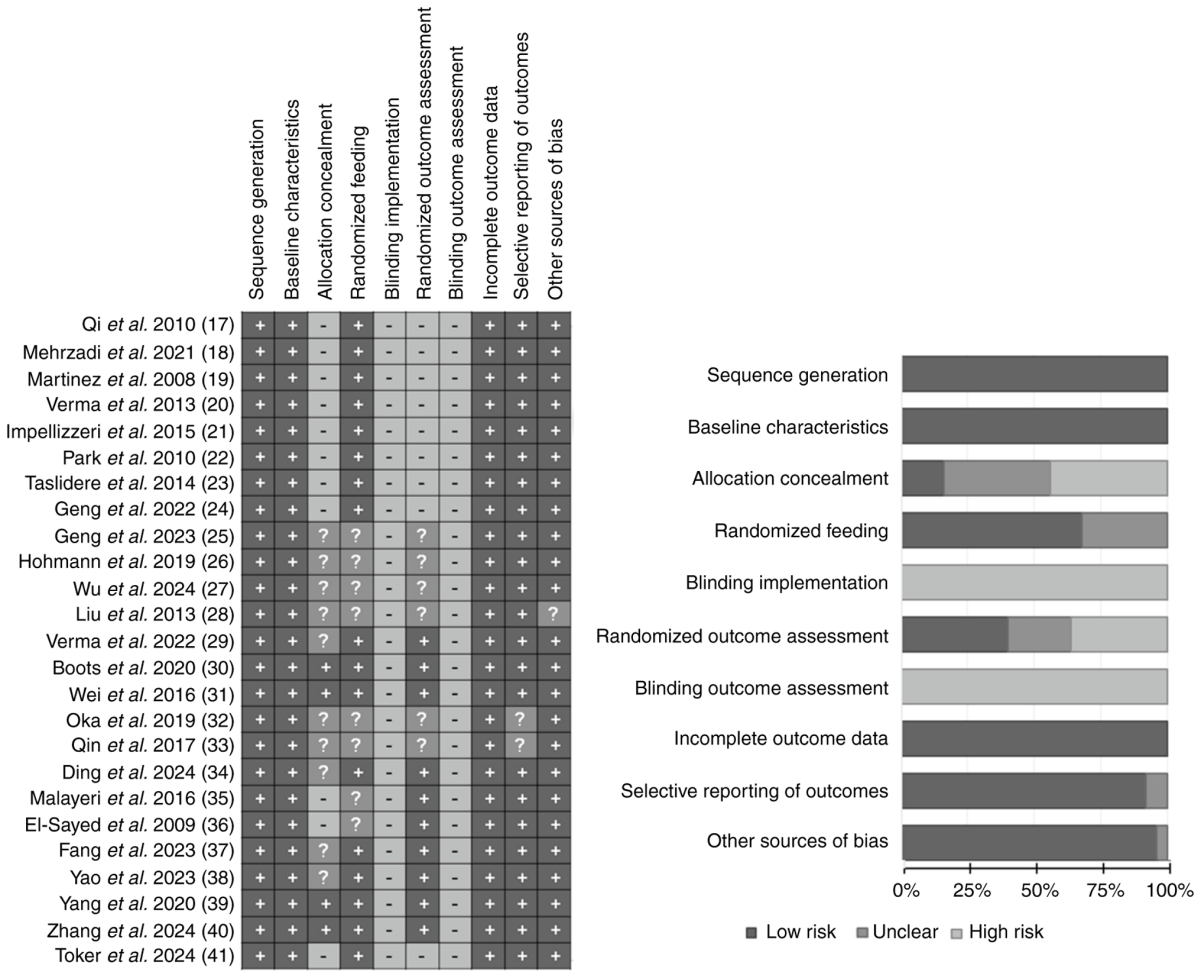
Risk of bias summary. A summary table of the authors' judgments for each risk of bias item for each study.

Among the 25 studies, the assessment results for sequence generation, baseline characteristics, incomplete outcome data, selective outcome reporting and other sources of bias were generally favorable, with 25, 25, 25, 23 and 24 studies rated as ‘low risk of bias’, respectively. No studies were scored as ‘high risk of bias’ for random housing. However, the assessments for blinding and blinding of outcome assessment were poor, with all 25 studies rated as ‘high risk of bias’. Overall, the studies performed well regarding randomization and outcome data, but there was notable bias in the implementation of blinding. The specific findings are summarized in Fig. 2. Due to the nature of study subjects and interventions, blinding of both participants and researchers was difficult and thus most of the studies did not report implementing double blinding.

### Research results

The present meta-analysis demonstrated that quercetin supplementation elicited notable changes across multiple physiological domains. As summarized in Table II, a significant increase in body weight was observed in the quercetin group compared with the control group (n=5; SMD=1.78; 95% CI, 0.72 to 2.84; I^2^=73%; P=0.0010). Similarly, lung index values were significantly reduced following quercetin intervention (n=4; SMD=-1.55; 95% CI, -3.04 to -0.05; I^2^=83%; P=0.04).

### Effect of quercetin on fibrosis-related markers

**Figure 3:**
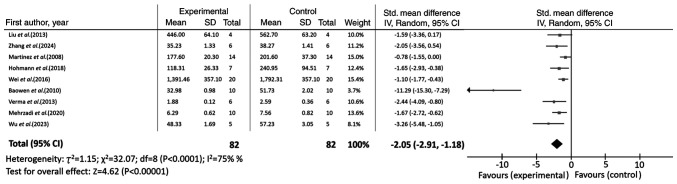
Forest plot illustrating weighted mean difference and 95% CI for the impact of quercetin on hydroxyproline. SD, standard deviation; IV, inverse variance.

Regarding fibrosis-related markers, quercetin administration resulted in marked improvements. Notably, hydroxyproline levels were significantly decreased (n=9; SMD=-2.05; 95% CI, -2.91 to -1.18; I^2^=75%; P<0.00001), as shown in Fig 3. Consistently, the Ashcroft score was also significantly lower in the quercetin group (n=7; SMD=-2.20; 95% CI, -3.21 to -1.18; I^2^=73%; P<0.0001). Furthermore, expression levels of Col I (n=4; SMD=-1.77; 95% CI, -2.85 to -0.69; I^2^=0%; P=0.001) and α-SMA (n=6; SMD=-2.25; 95% CI, -3.17 to -1.32; I^2^=53%; P<0.00001) were significantly suppressed, further supporting the anti-fibrotic effect of quercetin (Table II).

### Effect of quercetin on inflammatory markers

Analysis of inflammatory parameters revealed notable modulation by quercetin, which was evaluated through pro-inflammatory cytokines and inflammatory cells. The quercetin group exhibited significantly lower levels of key pro-inflammatory cytokines, including TNF-α (n=11; SMD=-1.73; 95% CI, -2.65 to -0.82; I^2^=80%; P=0.0002), IL-1β (n=6; SMD=-2.77; 95% CI, -3.55 to -2.00; I^2^=0%; P<0.00001, IL-6 (n=5; SMD=-1.45; 95% CI, -2.07 to -0.83; I^2^=0%; P<0.00001) and TGF-β1 (n=4; SMD=-2.68; 95% CI, -3.58 to -1.78; I^2^=0%; P<0.00001) (Table II).

**Figure 4:**
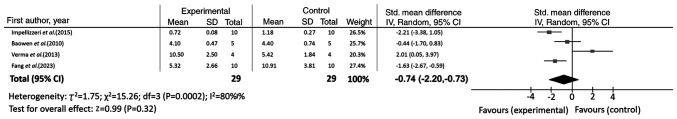
Forest plot illustrating weighted mean difference and 95% CI for the impact of quercetin on lymphocyte count. SD, standard deviation; IV, inverse variance.

With regards to inflammatory cell infiltration, quercetin supplementation significantly reduced counts of neutrophils (n=5; SMD=-3.73; 95% CI, -6.50 to -0.95; I^2^=89%; P=0.009), macrophages (n=7; SMD=-1.85; 95% CI, -3.36 to -0.35; I^2^=82%; P=0.02), eosinophils (n=3; SMD=-1.66; 95% CI, -3.25 to -0.06; I^2^=79%; P=0.04), leukocytes (n=3; SMD=-2.33; 95% CI, -3.89 to -0.77; I^2^=77%; P=0.003) and total cells (n=7; SMD=-1.32; 95% CI, -1.87 to -0.78; I^2^=37%; P<0.00001) (Table II). However, no significant effect was observed on lymphocyte count (n=4; SMD=-0.74; 95% CI, -2.20 to 0.73; I^2^=80%; P=0.32), as illustrated in Fig. 4.

### Effect of quercetin on oxidative stress markers

**Figure 5:**
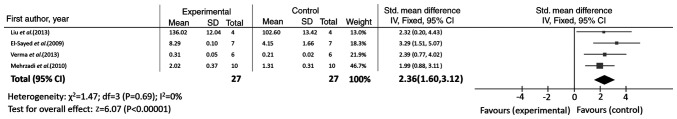
Forest plot illustrating weighted mean difference and 95% CI for the impact of quercetin on SOD activity. SOD, superoxide dismutase; SD, standard deviation; IV, inverse variance.

Quercetin supplementation significantly alleviated oxidative stress, as evidenced by the increased activities of antioxidant enzymes. Specifically, SOD activity was significantly enhanced (n=4; SMD=2.36; 95% CI, 1.60 to 3.12; I^2^=0%; P<0.00001), as shown in Fig. 5. CAT (n=4; SMD=1.99; 95% CI, 1.30 to 2.68; I^2^=41%; P<0.00001) activity and GSH levels were also significantly increased (n=6; SMD=1.93; 95% CI, 0.52 to 3.34; I^2^=85%; P=0.007) (Table II).

Conversely, quercetin significantly reduced the levels of biomarkers of oxidative damage, including MDA (n=7; SMD=-2.56; 95% CI, -3.46 to -1.66; I^2^=58%; P<0.00001), NO (n=4; SMD=-2.42; 95% CI, -3.63 to -1.21; I^2^=53%; P<0.0001) and TBARS (n=3; SMD=-1.15; 95% CI, -1.69 to -0.61; I^2^=9%; P<0.0001) (Table II).

### Meta-regression analysis for sources of heterogeneity

To explore potential sources of heterogeneity across the included studies, a meta-regression analysis was performed using four covariates: i) Animal model type (‘Model’); ii) fibrosis induction method (‘Pfinductiod’); iii) quercetin dosage (‘Quercetindose’); and i) intervention duration (‘Duration’).

The results indicated that quercetin dosage and intervention duration were the most influential factors contributing to heterogeneity across multiple outcome measures (Table III). Specifically, higher quercetin dosage was significantly associated with increased levels of the antioxidant marker GSH [unstandardized regression coefficients (coef.)=0.107; P=0.035] and lymphocyte count (coef.=0.040; P=0.035), decreased total inflammatory cell count (coef.=-0.025; P=0.027) and leukocyte cell count (coef.=-0.030; P=0.002). A longer intervention duration was significantly associated with increased CAT activity (coef.=0.101; P=0.044) and elevated GSH levels (coef.=0.228; P=0.017).

The choice of fibrosis induction method emerged as a significant source of heterogeneity for macrophage infiltration (coef.=-0.947; P=0.002) and changes in body weight (coef.=2.915; P=0.007). Conversely, the type of animal model was significantly associated with variations in MDA (coef.=0.468; P=0.003) and TNF-α levels (coef.=0.584; P=0.009).

For numerous outcomes, including inflammatory cytokines (IL-1β and IL-6) and fibrosis markers (COL I and α-SMA), none of the examined covariates demonstrated a significant moderating effect (all P>0.05), suggesting that other unmeasured factors likely contributed to the observed heterogeneity.

### Publication bias

**Figure 6:**
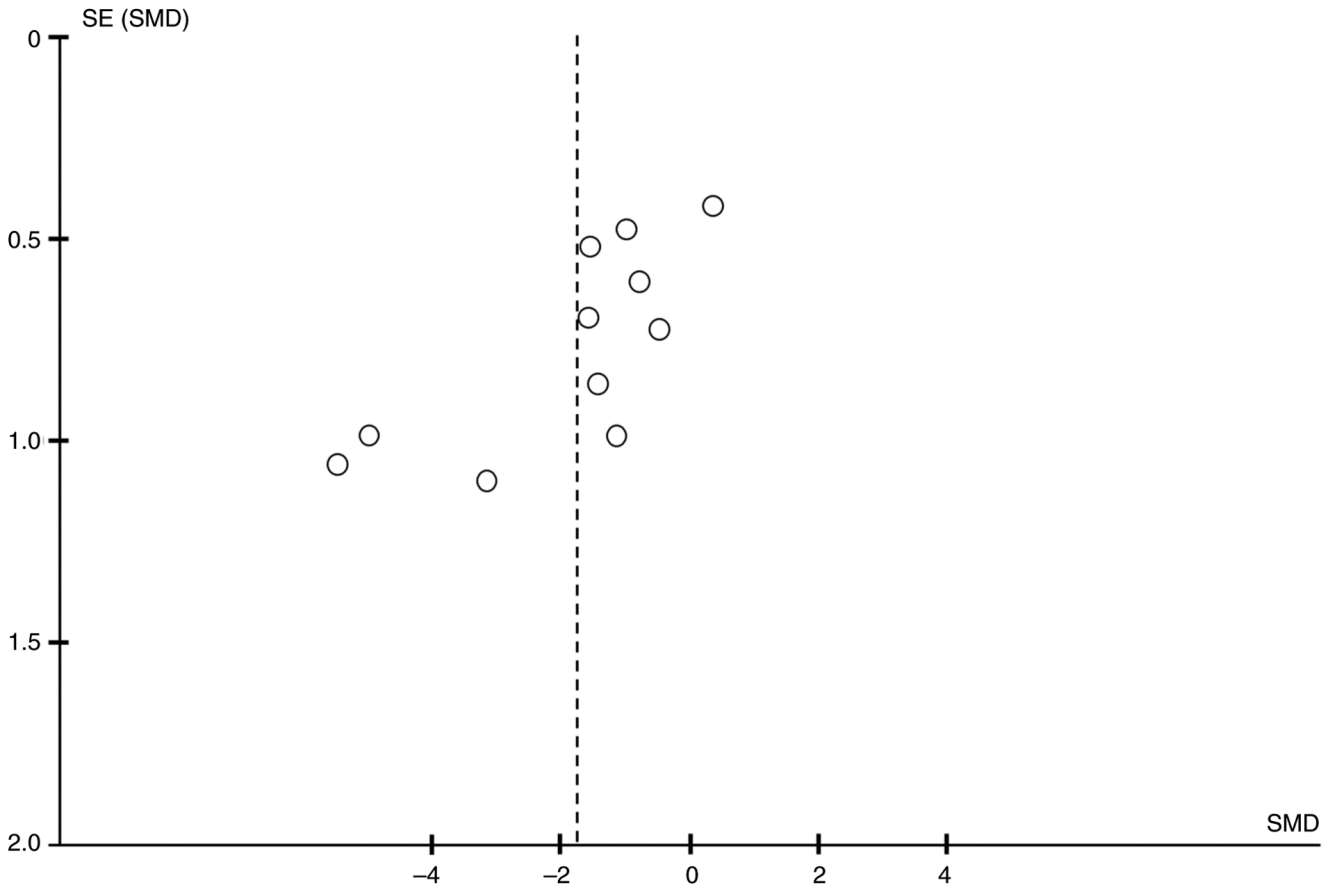
Funnel plot for TNF-α demonstrated a roughly symmetrical distribution (n=11). SE, standard error; SMD, standardized mean difference.

The potential for publication bias was systematically evaluated for all outcomes using both Egger's linear regression test and Begg's rank correlation test (Table IV). The visual inspection of funnel plots indicated general symmetry for numerous outcomes (for example, TNF-α; Fig. 6). In accordance with methodological recommendations (including the Cochrane Handbook), statistical tests for funnel plot asymmetry, such as Egger's test, are only recommended when a meta-analysis contains ≥10 studies. Among all outcomes in the present analysis, TNF-α exhibited the largest number of studies (n=11), meeting this minimum threshold. Therefore, funnel plots and statistical tests for this outcome were selectively performed and reported to provide a meaningful assessment. The statistical tests demonstrated that no significant publication bias was detected for the majority of outcomes (all P>0.05).

However, significant publication bias was identified for four specific outcomes. Egger's test yielded statistically significant results for hydroxyproline (t=-4.60; P=0.002), Col I (t=-5.14; P=0.036), TNF-α (t=-3.45; P=0.007) and GSH (t=4.32; P=0.012). The findings from Begg's test further supported the presence of a significant bias for hydroxyproline (z=-2.50; P=0.012) and Col I (z=-2.04; P=0.042), while the result for TNF-α was of borderline statistical significance (P=0.052), and no significant bias was detected for GSH (P=0.091) using this method.

## Discussion

The present meta-analysis evaluated the therapeutic potential of quercetin in experimental models of PF to explore potential sources of heterogeneity in its efficacy. The present comprehensive analysis indicated that quercetin intervention may exert regulatory effects across multiple physiological processes, including body weight recovery, attenuation of fibrosis progression, suppression of inflammatory responses and reduction of oxidative stress. These findings are consistent with previous experimental studies (42-44).

Previous studies have suggested that quercetin may inhibit collagen synthesis through modulation of the TGF-β1/Smad signaling pathway (45,46), while also promoting collagen degradation by regulating the MMP/TIMP balance; thereby demonstrating anti-fibrotic effects. This is reflected in reduced levels of hydroxyproline, Col I, α-SMA and lower Ashcroft scores. However, meta-regression analysis indicated that the type of animal model may be a notable source of heterogeneity in fibrosis markers, suggesting that the genetic backgrounds of different animal strains may influence treatment responsiveness.

With regards to anti-inflammatory mechanisms, the present study observed that quercetin may reduce the levels of pro-inflammatory cytokines, including TNF-α, IL-1β, IL-6 and TGF-β1, as suggested in the literature through inhibition of the NF-κB and MAPK signaling pathways (47-49). Notably, the method of fibrosis induction markedly influenced the degree of inflammatory cell infiltration, indicating that different induction methods (for example, bleomycin vs. silica) may activate distinct inflammatory pathways, thereby perhaps contributing to variability in treatment outcomes.

In relation to its antioxidant effects, quercetin may enhance the activities of SOD, CAT and GSH through activation of the nuclear factor erythroid 2-related factor 2 (Nrf2)/Kelch-like ECH-associated protein 1/antioxidant response element pathway (50,51). Meta-regression analysis revealed a positive association between quercetin dosage and GSH levels and intervention duration was also notably associated with CAT activity and GSH, suggesting that its antioxidant effects may be dose- and time-dependent.

Interactions among experimental design parameters add complexity to the assessment of efficacy. The present study found that both animal model selection and induction method jointly influence treatment outcomes. For example, the present data indicate that different induction methods may produce markedly distinct pathological phenotypes across animal strains. Furthermore, we hypothesize that there may be an interaction between quercetin dosage and intervention duration, suggesting that long-term high-dose treatment could yield synergistic effects, although this hypothesis warrants further validation.

It should be noted that the high heterogeneity observed in the present study may affect the interpretation of results. Although several indicators, including hydroxyproline, lung index, TNF-α and neutrophil count, exhibited high heterogeneity (I^2^>75%), this variation largely reflects methodological diversity across studies rather than fundamental differences in treatment effects. Importantly, meta-regression analysis demonstrated the beneficial therapeutic effect of quercetin across all studies, with heterogeneity primarily influencing the magnitude rather than the direction of the effect.

Notably, quercetin did not demonstrate a notable effect on lymphocyte count, contrasting with its pronounced effects on innate immune cells. This may indicate differential regulatory activity on innate compared with adaptive immune responses. For other outcomes with high heterogeneity but statistical significance, the results suggested context-dependent variability.

Moreover, for certain indicators (for example, IL-1β, IL-6, Col I or α-SMA), none of the covariates examined showed notable influence, indicating that other unmeasured sources of heterogeneity, such as animal age, sex differences or analytical method variations, may be present.

The present meta-analysis provided a comprehensive and quantitative summary of the current preclinical evidence regarding the therapeutic effects of quercetin in experimental PF. One of the primary strengths is the integration of data from 25 independent studies, which enhances the statistical power and allows for a more robust estimation of treatment effects across multiple outcome domains, including fibrotic, inflammatory and oxidative stress parameters. The application of random-effects meta-analysis and meta-regression analysis further strengthens the present study by accounting for between-study heterogeneity and exploring the influence of key experimental variables, such as dosage, duration, animal model and induction method. This approach not only increases the reliability of the findings but also helps identify subtle and consistent treatment effects that may not be apparent in individual studies. Moreover, the present study offers novel insights into the context-dependent efficacy of quercetin and highlights potential sources of heterogeneity, thereby contributing to the optimization of future preclinical research design.

Several limitations should be considered when interpreting the present results. First, the inclusion of studies with varying methodological quality, particularly in areas such as randomization and blinding, a common issue in animal studies, may introduce bias and affect the validity of pooled effect estimates. Second, the presence of notable heterogeneity, although partially explained by meta-regression, remains a concern, as unmeasured factors such as animal age, sex and specific analytical protocols may contribute to variability. Third, the reliance on data extracted from figures in certain studies, despite efforts to obtain original datasets, may have introduced inaccuracies in measurement. Finally, all included studies were conducted in animal models, which inherently limits the direct translatability of the findings to human patients. These limitations, however, are reflective of broader challenges in preclinical meta-research rather than specific flaws in the current methodology.

The present meta-analysis indicated that quercetin administration was associated with notable improvements in fibrotic, inflammatory and oxidative stress parameters, potentially through the modulation of key pathways such as TGF-β1/Smad, NF-κB and Nrf2 signaling.

However, these findings must be interpreted with caution due to the inherent limitations of the included preclinical studies. The notable methodological heterogeneity, variability in experimental design and lack of clinical validation, all of which are common in animal research, undermine the robustness of the results and render their translational relevance to human disease uncertain. Consequently, the implications of the present analysis should be considered hypothesis-generating rather than definitive.

To strengthen the evidence, future investigations should prioritize: i) Standardizing experimental protocols to minimize heterogeneity; ii) performing rigorous dose-response and time-course studies; iii) validating these findings across a broader range of PF models; and iv) enhancing data transparency and reproducibility.

In summary, while the present meta-analysis highlights the promising therapeutic potential of quercetin and provides a rationale for further mechanistic investigation, its ultimate clinical value can only be established through well-designed future clinical trials.

## Supplementary Material

Search strategy for PubMed.

Search strategy for Cochrane Library.

Search strategy for Embase.

Search strategy for Ovid.

Search strategy for Web of Science.

## Figures and Tables

**Table I:** Basic characteristics of the included studies.

First author, year	Country	Age	Total, n	Species; model	PF induction method	Quercetin dose (route)	Duration	Outcome	(Refs.)
Baowen *et al*, 2010	China	N	40	Rats; Sprague-Dawley	Bleomycin (intratracheal)	5 mg/kg (intravenous)	28 days	TNF-α, IL-1β, IL-6, total cell numbers, lymphocytes, neutrophils, macrophages and hydroxyproline	(17)
Mehrzadi *et al*, 2021	Iran	N	20	Male rats; Wistar	Bleomycin (intratracheal)	75 mg/kg (oral gavage)	28 days	TNF-α, IL-6, GSH, CAT, NO, TBARS, Ashcroft score, lung index and hydroxyproline	(18)
Martinez *et al*, 2008	Brazil	N	28	Male hamsters; *Mesocricetus* *auratus*	Bleomycin (intratracheal)	30 mg/kg (intraperitoneal)	14 days	GSH, TBARS and hydroxyproline	(19)
Verma *et al*, 2013	India	10-12 weeks	20	Male rats; Wistar	Bleomycin (intratracheal)	100 mg/kg (oral)	20 days	TNF-α, CAT, SOD, MDA, weight, hydroxyproline, total cell numbers, neutrophils, macrophages, eosinophil and lymphocytes	(20)
Impellizzeri *et al*, 2015	Italy	N	20	Male mice; CD1(ICR)	Bleomycin (intratracheal)	10 mg/kg (oral)	7 days	Ashcroft score, total cell numbers, weight, neutrophils, lymphocytes, macrophages, eosinophil and leucocytes	(21)
Park *et al*, 2010	Korea	N	8	Male rats; Sprague-Dawley	Paraquat (intratracheal)	50 mg/kg (intraperitoneal)	14 days	GSH, MDA and NO	(22)
Taslidere *et al*, 2014	Turkey	3-4 months	14	Albino female rats; Wistar	CCl_4_ (intraperitoneal)	25 mg/kg (intraperitoneal)	10 days	GSH, MDA and CAT	(23)
Geng *et* al, 2022	China	6-8 weeks	20	Female mice; C57BL/6	SiO_2_ (intratracheal)	100 mg/kg (oral gavage)	28 days	TNF-α, IL-1β, IL-6 and Col I	(24)
Geng *et al*, 2023	China	8 weeks	10	Male mice; C57BL/6	SiO_2_ (intratracheal)	100 mg/kg (intraperitoneal)	28 days	α-SMA and Col I	(25)
Hohmann *et al*, 2019	America	>12 months	14	Male and female mice; C57BL/6	Bleomycin (intratracheal)	30 mg/kg (intraperitoneal)	21 days	Weight and hydroxyproline	(26)
Wu *et al*, 2024	China	8 weeks	10	Female rats; Wistar	Bleomycin (intratracheal)	75 mg/kg (oral)	28 days	α-SMA, Col I, hydroxyproline and weight	(27)
Liu *et al*, 2013	China	6-8 weeks	46	Female mice; C57BL/6	Cobalt-60γ radiation (16 Gy; thoracic irradiation)	5 mg/kg (intraperitoneal)	24 weeks	α-SMA, TNF-α, TGF-β1, SOD, MDA, total cell numbers, hydroxy proline and Ashcroft score	(28)
Verma *et al*, 2022	India	8-10 weeks	12	Female mice; C57BL/6	γ radiation (12 Gy) (thoracic irradiation)	10 mg/kg (intramuscularly)	16 weeks	α-SMA, TNF-α, IL-1β, IL-6, TGF-β1, MDA, NO, lung index, macrophages, total cell numbers, Ashcroft score and leucocytes	(29)
Boots *et al*, 2020	Germany	10-12 weeks	23	Male and female mice; C57BL/6	Bleomycin (pharyngeal administration)	200 mg/kg (oral)	21 days	TNF-α and weight	(30)
Wei *et al*, 2016	China	18 weeks	40	Male rats; Sprague-Dawley	Bleomycin (intraperitoneal)	3 mg/kg (intraperitoneal)	36 days	Ashcroft score, hydroxyproline and MDA	(31)
Oka *et al*, 2019	Nigeria	N	12	Female rats; Wistar	Amiodarone (intratracheal)	20 mg/kg (oral)	21 days	GSH, CAT, total cell numbers and macrophages	(32)
Qin *et al*, 2017	China	5-6 weeks	24	Male rats; Wistar	X-ray (15Gy) (pulmonary apex irradiation)	100 mg/kg (inhaled)	4 months	Leucocytes	(33)
Ding *et al*, 2024	China	6-8 weeks	12	Male mice; C57BL/6	PM2:5 (intratracheal)	50 mg/kg (oral gavage)	60 days	TNF-α, IL-1β, IL-6, TGF-β1, Col I, Ashcroft score and lung index	(34)
Malayeri *et al*, 2016	Iran	N	20	Male rats; Sprague-Dawley	Bleomycin (intratracheal)	50 mg/kg (intraperitoneal)	28 days	TNF-α	(35)
El-Sayed *et al*, 2009	Egypt	N	14	Albino male rats; N	Paraquat (intraperitoneal)	50 mg/kg (p.o.)	21 days	GSH, SOD, NO and TBARS	(36)
Fang *et al*, 2023	China	6 weeks	20	Male mice; C57BL/6	10 mg/ml OVA and alum adjuvant (intraperitoneal) OVA (inhaled)	30 mg/kg (gavage)	21 days	TGF-β1, α-SMA, total cell numbers, neutrophils, lymphocytes, eosinophil and macrophages	(37)
Yao *et al*, 2023	China	N	20	Rats; Sprague-Dawley	Silica suspension (intratracheal)	2 mg/kg (intratracheal)	28 days	TNF-α, IL-1β, α-SMA, SOD, weight and lung index	(38)
Yang *et al*, 2020	China	6-8 weeks	24	Male mice; BALB/c	Cigarette smoke (inhaled)	50 mg/kg (intraperitoneal)	12 weeks	TNF-α, IL-1β, total cell numbers, neutrophils and macrophages	(39)
Zhang *et al*, 2024	China	6-8 weeks	12	Male mice; C57BL/6	Bleomycin (intratracheal)	50 mg/kg (intragastric administration)	3 weeks	Ashcroft score and hydroxyproline	(40)
Toker *et al*, 2024	Turkey	N	16	Albino male rats; Wistar	Bleomycin (intratracheal)	50 mg/kg (intraperitoneal)	21 days	α-SMA	(41)

PF, pulmonary fibrosis; TBARS, thiobarbituric acid reactive substances; Col I, collagen I; α-SMA, α-smooth muscle actin; GSH, glutathione; CAT, catalase; SOD, superoxide dismutase; SiO_2_, silicon dioxide; MDA, malondialdehyde; NO, nitric oxide; p.o., per os; N, none.

**Table II:** Results of the meta-analysis for each outcome indicator.

	Heterogeneity test results			Meta-analysis results		
Outcome indicator	I^2^, %	P-value	Effect models	SMD	95% CI	P-value
Weight	73	0.005	Random	1.78	(0.72, 2.84)	0.001
Lung index	83	0.0005	Random	-1.55	(-3.04, -0.05)	0.040
TBARS	9	0.330	Fixed	-1.15	(-1.69, -0.61)	<0.0001
Ashcroft score	73	0.001	Random	-2.20	(-3.21, -1.18)	<0.0001
Hydroxyproline	75	<0.0001	Random	-2.05	(-2.91, -1.18)	<0.00001
Col I	0	0.710	Fixed	-1.77	(-2.85, -0.69)	0.001
α-SMA	53	0.060	Random	-2.25	(-3.17, -1.32)	<0.00001
TNF-α	80	<0.0001	Random	-1.73	(-2.65, -0.82)	0.0002
IL-1β	0	0.410	Fixed	-2.77	(-3.55, -2.00)	<0.00001
IL-6	0	0.410	Fixed	-1.45	(-2.07, -0.83)	<0.00001
TGF-β1	0	0.980	Fixed	-2.68	(-3.58, -1.78)	<0.00001
GSH	85	<0.00001	Random	1.93	(0.52, 3.34)	0.007
CAT	41	0.170	Fixed	1.99	(1.30, 2.68)	<0.00001
SOD	0	0.690	Fixed	2.36	(1.60, 3.12)	<0.00001
MDA	58	0.030	Random	-2.56	(-3.46, -1.66)	<0.00001
NO	53	0.090	Random	-2.42	(-3.63, -1.21)	<0.0001
Neutrophils	89	<0.00001	Random	-3.73	(-6.50, -0.95)	0.009
Lymphocytes	80	0.002	Random	-0.74	(-2.20, 0.73)	0.320
Macrophages	82	<0.00001	Random	-1.85	(-3.36, -0.35)	0.020
Eosinophil	79	0.009	Random	-1.66	(-3.25, -0.06)	0.040
Total cell numbers	37	0.150	Fixed	-1.32	(-1.87, -0.78)	<0.00001
Leucocytes	77	0.010	Random	-2.33	(-3.89, -0.77)	0.003

SMD, standardized mean difference; TBARS, thiobarbituric acid reactive substances; Col I, collagen I; α-SMA, α-smooth muscle actin; GSH, glutathione; CAT, catalase; SOD, superoxide dismutase; MDA, malondialdehyde; NO, nitric oxide.

**Table III:** Meta-regression analysis of potential moderators for various outcome measures.

Outcome measure	Moderator	Coefficient	SE	z-value	P-value	95% CI
α-SMA	Model	-0.171	0.342	-0.50	0.618	-0.841 to 0.500
	Pfinductiod	-0.293	0.273	-1.07	0.284	-0.829 to 0.243
	Quercetindose	-0.009	0.020	-0.46	0.642	-0.048 to 0.030
	Duration	0.018	0.012	1.54	0.124	-0.005 to 0.042
CAT	Model	-1.389	0.799	-1.74	0.082	-2.956 to 0.178
	Pfinductiod	-0.082	0.284	-0.29	0.773	-0.638 to 0.475
	Quercetindose	-0.007	0.018	-0.37	0.709	-0.042 to 0.029
	Duration	0.101	0.050	2.02	0.044^a^	0.003 to 0.198
Col I	Model	-0.442	0.355	-1.24	0.214	-1.138 to 0.255
	Pfinductiod	-0.302	0.366	-0.83	0.409	-1.020 to 0.415
	Quercetindose	-0.011	0.034	-0.33	0.739	-0.077 to 0.055
	Duration	-0.022	0.044	-0.51	0.612	-0.108 to 0.063
GSH	Model	0.479	0.455	1.05	0.292	-0.413 to 1.371
	Pfinductiod	-0.030	0.547	-0.05	0.957	-1.102 to 1.042
	Quercetindose	0.107	0.051	2.11	0.035^a^	0.008 to 0.207
	Duration	0.228	0.096	2.38	0.017^a^	0.040 to 0.416
Hydroxyproline	Model	0.475	0.507	0.94	0.349	-0.519 to 1.468
	Pfinductiod	0.475	1.405	0.34	0.735	-2.279 to 3.229
	Quercetindose	0.009	0.029	0.33	0.744	-0.047 to 0.066
	Duration	0.005	0.019	0.26	0.798	-0.033 to 0.043
IL-1β	Model	-0.204	0.212	-0.96	0.336	-0.621 to 0.212
	Pfinductiod	-0.119	0.232	-0.51	0.610	-0.574 to 0.336
	Quercetindose	-0.006	0.016	-0.39	0.695	-0.039 to 0.026
	Duration	-0.018	0.015	-1.20	0.230	-0.046 to 0.011
IL-6	Model	-0.153	0.160	-0.96	0.338	-0.466 to 0.160
	Pfinductiod	-0.094	0.194	-0.48	0.629	-0.474 to 0.287
	Quercetindose	-0.019	0.014	-1.31	0.190	-0.046 to 0.009
	Duration	-0.006	0.010	-0.58	0.561	-0.025 to 0.013
MDA	Model	0.468	0.160	2.93	0.003^a^	0.155 to 0.782
	Pfinductiod	0.164	0.354	0.46	0.643	-0.529 to 0.858
	Quercetindose	-0.007	0.015	-0.45	0.653	-0.037 to 0.023
	Duration	0.012	0.008	1.55	0.121	-0.003 to 0.027
NO	Model	-0.386	0.198	-1.95	0.051	-0.775 to 0.002
	Pfinductiod	-0.586	0.546	-1.07	0.284	-1.656 to 0.485
	Quercetindose	-0.016	0.053	-0.31	0.760	-0.119 to 0.087
	Duration	0.008	0.023	0.33	0.740	-0.037 to 0.053
Ashcroft score	Model	0.759	0.507	1.50	0.135	-0.236 to 1.753
	Pfinductiod	0.565	0.650	0.87	0.385	-0.709 to 1.838
	Quercetindose	-0.059	0.038	-1.53	0.125	-0.133 to 0.016
	Duration	0.015	0.017	0.88	0.379	-0.019 to 0.049
SOD	Model	0.210	0.163	1.29	0.198	-0.110 to 0.530
	Pfinductiod	0.378	0.311	1.22	0.224	-0.232 to 0.989
	Quercetindose	-0.0003	0.014	-0.02	0.984	-0.027 to 0.027
	Duration	0.0004	0.008	0.05	0.960	-0.015 to 0.016
TBARS	Model	-0.005	0.194	-0.02	0.981	-0.385 to 0.376
	Pfinductiod	-0.129	0.314	-0.41	0.682	-0.745 to 0.487
	Quercetindose	-0.044	0.027	-1.59	0.112	-0.097 to 0.010
	Duration	-0.070	0.046	-1.54	0.124	-0.160 to 0.019
TGF-β1	Model	0.451	0.921	0.49	0.624	-1.353 to 2.25
	Pfinductiod	0.155	0.320	0.48	0.628	-0.473 to 0.783
	Quercetindose	0.014	0.032	0.43	0.668	-0.048 to 0.076
	Duration	-0.004	0.008	-0.48	0.631	-0.020 to 0.012
TNF-α	Model	0.584	0.223	2.62	0.009	0.147 to 1.021
	Pfinductiod	0.136	0.309	0.44	0.660	-0.469 to 0.741
	Quercetindose	0.015	0.008	1.83	0.067	-0.001 to 0.031
	Duration	0.013	0.012	1.05	0.295	-0.011 to 0.036
Leukocyte	Model	0.953	0.398	2.39	0.017	0.172 to 1.734
	Pfinductiod	-0.653	1.114	-0.59	0.558	-2.837 to 1.531
	Quercetindose	-0.030	0.010	-3.07	0.002	-0.049 to -0.011
	Duration	-0.013	0.020	-0.67	0.501	-0.052 to 0.025
Lung index	Model	0.615	0.444	1.38	0.166	-0.256 to 1.485
	Pfinductiod	0.581	0.726	0.80	0.423	-0.842 to 2.004
	Quercetindose	0.052	0.043	1.22	0.223	-0.032 to 0.137
	Duration	0.027	0.031	0.89	0.375	-0.033 to 0.088
Macrophages	Model	-0.712	0.634	-1.12	0.261	-1.956 to 0.531
	Pfinductiod	-0.947	0.312	-3.03	0.002	-1.559 to -0.335
	Quercetindose	0.039	0.035	1.11	0.266	-0.030 to 0.109
	Duration	-0.009	0.033	-0.26	0.796	-0.074 to 0.057
Lymphocytes	Model	-0.674	0.570	-1.18	0.237	-1.791 to 0.444
	Pfinductiod	-0.290	0.511	-0.57	0.571	-1.291 to 0.712
	Quercetindose	0.040	0.019	2.11	0.035	0.003 to 0.077
	Duration	0.096	0.140	0.68	0.495	-0.179 to 0.371
Eosinophils	Model	-0.790	0.636	-1.24	0.214	-2.038 to 0.457
	Pfinductiod	-0.102	0.525	-0.19	0.846	-1.132 to 0.927
	Quercetindose	0.033	0.010	3.17	0.002	0.013 to 0.053
	Duration	0.140	0.142	0.99	0.324	-0.139 to 0.419
Body weight	Model	-0.350	0.444	-0.79	0.430	-1.220 to 0.520
	Pfinductiod	2.915	1.076	2.71	0.007	0.806 to 5.024
	Quercetindose	-0.023	0.012	-2.01	0.044	-0.046 to -0.001
	Duration	0.018	0.092	0.20	0.843	-0.162 to 0.198
Total cell count	Model	-0.032	0.269	-0.12	0.905	-0.560 to 0.496
	Pfinductiod	-0.280	0.197	-1.42	0.156	-0.667 to 0.106
	Quercetindose	-0.025	0.011	-2.22	0.027^a^	-0.047 to -0.003
	Duration	0.007	0.007	0.91	0.362	-0.008 to 0.021
Neutrophils	Model	-0.510	0.940	-0.54	0.587	-2.353 to 1.332
	Pfinductiod	0.234	0.680	0.34	0.731	-1.098 to 1.566
	Quercetindose	-0.032	0.053	-0.61	0.543	-0.136 to 0.072
	Duration	0.031	0.068	0.46	0.645	-0.102 to 0.165

^a^P<0.05. Model type (‘Model’) and induction method (‘Pfinductiod’) are categorical moderators. Quercetin dose (‘Quercetindose’) and duration (‘Duration’) are continuous moderators. SE, standard error; α-SMA, alpha-smooth muscle actin; CAT, catalase; COL I, collagen type I; GSH, reduced glutathione.

**Table IV:** Results of the Begg's and Egger's tests for assessment of potential publication bias.

		Begg's test		Egger's test	
Outcome measure	n	z-value	P-value	t-value	P-value
Boby weight	5	1.96	0.050	2.25	0.110
Lung index	4	-0.68	0.497	-1.84	0.207
TBARS	3	-0.52	0.602	-2.10	0.283
Ashcroft score	7	-1.35	0.176	-1.87	0.120
Hydroxyproline content	9	-2.50	0.012^a^	-4.60	0.002^b^
Col I	4	-2.04	0.042^a^	-5.14	0.036^a^
α-SMA	6	-1.69	0.091	-1.04	0.357
TNF-α	11	-1.95	0.052	-3.45	0.007^b^
IL-1β	6	-0.94	0.348	-0.81	0.463
IL-6	5	-1.47	0.142	-1.78	0.173
TGF-β1	4	0.68	0.497	0.20	0.860
GSH	6	1.69	0.091	4.32	0.012^a^
CAT	4	1.36	0.174	1.22	0.347
SOD	4	0.68	0.497	1.18	0.359
MDA	7	0.45	0.652	-0.27	0.801
NO	4	-1.36	0.174	-1.88	0.200
Neutrophils	5	-1.47	0.142	-1.90	0.154
Lymphocytes	4	1.36	0.174	2.94	0.099
Macrophages	7	-0.15	0.881	-0.58	0.586
Eosinophils	3	0.52	0.602	0.33	0.800
Total cell count	7	-1.35	0.176	-2.20	0.079
Leukocyte count	3	-1.57	0.117	-1.08	0.475

^a^P<0.05 and

^b^P<0.01. n, number of studies included in the analysis; Col I, collagen I; α-SMA, α-smooth muscle actin; GSH, glutathione; CAT, catalase; SOD, superoxide dismutase; MDA, malondialdehyde; NO, nitric oxide; TBARS, thiobarbituric acid reactive substances.

## Data Availability

The data generated in the present study may be requested from the corresponding author.
