# Supplementary material for: Therapeutic efficacy of quercetin in experimental pulmonary fibrosis: A meta-analysis
Source: Exp Ther Med. 2025 Dec 22;31(2):59. doi: 10.3892/etm.2025.13054 (PMC12766670; doi:10.3892/etm.2025.13054)
Supplement: Search strategy for PubMed. [file Supplementary_Data.pdf]

Table SI. Search strategy for PubMed.

**Search no.**	**PubMed**
#1	((Pulmonary fibrosis[MeSH Terms]) OR (Idiopathic Pulmonary Fibrosis[MeSH Terms])) OR (Asbestosis[MeSH Terms])
#2	((((((((((((((((((((((((((((((((((Pulmonary fibrosis) OR (Idiopathic Pulmonary Fibrosis)) OR (Asbestosis)) OR (Fibroses, Pulmonary)) OR (Fibrosis, Pulmonary)) OR (Pulmonary Fibroses)) OR (Alveolitis, Fibrosing)) OR (Alveolitides, Fibrosing)) OR (Fibrosing Alveolitides)) OR (Fibrosing Alveolitis)) OR (Idiopathic Diffuse Interstitial Pulmonary Fibrosis)) OR (Idiopathic Pulmonary Fibroses)) OR (Pulmonary Fibroses, Idiopathic)) OR (Cryptogenic Fibrosing Alveolitis)) OR (Cryptogenic Fibrosing Alveolitides)) OR (Fibrosing Alveolitides, Cryptogenic)) OR (Pulmonary Fibrosis, Idiopathic)) OR (Fibrosing Alveolitis, Cryptogenic)) OR (Fibrocystic Pulmonary Dysplasia)) OR (Dysplasia, Fibrocystic Pulmonary)) OR (Fibrocystic Pulmonary Dysplasias)) OR (Pulmonary Dysplasia, Fibrocystic)) OR (Idiopathic Fibrosing Alveolitis, Chronic Form)) OR (Familial Idiopathic Pulmonary Fibrosis)) OR (Idiopathic Pulmonary Fibrosis, Familial)) OR (Usual Interstitial Pneumonia)) OR (Interstitial Pneumonia, Usual)) OR (Usual Interstitial Pneumonias)) OR (Interstitial Pneumonitis, Usual)) OR (Pneumonitides, Usual Interstitial)) OR (Pneumonitis, Usual Interstitial)) OR (Usual Interstitial Pneumonitides)) OR (Usual Interstitial Pneumonitis)) OR (Asbestoses)) OR (Pulmonary Fibrosis - from Asbestos Exposure)) OR (Idiopathic Interstitial Pneumonitis - from Asbestos Exposure)
#3	(#1) OR (#2)
#4	Quercetin [MeSH Terms]
#5	((Quercetin) OR (3,3',4',5,7-Pentahydroxyflavone)) OR (Dikvertin)
#6	(#4) OR (#5)
#7	(#3) AND (#6)

Table SII. Search strategy for Cochrane Library.

Search no.	Cochrane Library
#1	(Pulmonary fibrosis):ti,ab,kw OR (Idiopathic Pulmonary Fibrosis):ti,ab,kw OR (Asbestosis):ti,ab,kw OR (Fibroses, Pulmonary):ti,ab,kw OR (Fibrosis, Pulmonary):ti,ab,kw
#2	(Pulmonary fibrosis):ti,ab,kw OR (Idiopathic Pulmonary Fibrosis):ti,ab,kw OR (Asbestosis):ti,ab,kw OR (Fibroses, Pulmonary):ti,ab,kw OR (Fibrosis, Pulmonary):ti,ab,kw
#3	(Idiopathic Diffuse Interstitial Pulmonary Fibrosis):ti,ab,kw OR (Idiopathic Pulmonary Fibroses):ti,ab,kw OR (Pulmonary Fibroses, Idiopathic):ti,ab,kw OR (Cryptogenic Fibrosing Alveolitis):ti,ab,kw OR (Cryptogenic Fibrosing Alveolitides):ti,ab,kw
#4	(Idiopathic Diffuse Interstitial Pulmonary Fibrosis):ti,ab,kw OR (Idiopathic Pulmonary Fibroses):ti,ab,kw OR (Pulmonary Fibroses, Idiopathic):ti,ab,kw OR (Cryptogenic Fibrosing Alveolitis):ti,ab,kw OR (Cryptogenic Fibrosing Alveolitides):ti,ab,kw
#5	(Idiopathic Diffuse Interstitial Pulmonary Fibrosis):ti,ab,kw OR (Idiopathic Pulmonary Fibroses):ti,ab,kw OR (Pulmonary Fibroses, Idiopathic):ti,ab,kw OR (Cryptogenic Fibrosing Alveolitis):ti,ab,kw OR (Cryptogenic Fibrosing Alveolitides):ti,ab,kw
#6	(Usual Interstitial Pneumonia):ti,ab,kw OR (Interstitial Pneumonia, Usual):ti,ab,kw OR (Usual Interstitial Pneumonias):ti,ab,kw OR (Interstitial Pneumonitis, Usual):ti,ab,kw OR (Pneumonitides, Usual Interstitial):ti,ab,kw
#7	(Pneumonitis, Usual Interstitial):ti,ab,kw OR (Usual Interstitial Pneumonitides):ti,ab,kw OR (Usual Interstitial Pneumonitis):ti,ab,kw OR (Asbestoses):ti,ab,kw OR (Pulmonary Fibrosis from Asbestos Exposure):ti,ab,kw
#8	(Pneumonitis, Usual Interstitial):ti,ab,kw OR (Usual Interstitial Pneumonitides):ti,ab,kw OR (Usual Interstitial Pneumonitis):ti,ab,kw OR (Asbestoses):ti,ab,kw OR (Pulmonary Fibrosis from Asbestos Exposure):ti,ab,kw
#9	#1 OR #2 OR #3 OR #4 OR #5 OR #6 OR #7OR #8
#10	(Quercetin):ti,ab,kw OR (3,3',4',5,7 Pentahydroxyflavone):ti,ab,kw OR (Dikvertin):ti,ab,kw
#11	(#9) AND (#10)

Table SIII. Search strategy for Embase.

Search no.	Embase
#1	'pulmonary fibrosis'/exp OR 'pulmonary fibrosis' OR (pulmonary AND ('fibrosis'/exp OR fibrosis)) OR 'idiopathic pulmonary fibrosis'/exp OR 'idiopathic pulmonary fibrosis' OR (idiopathic AND pulmonary AND ('fibrosis'/exp OR fibrosis)) OR 'asbestosis'/exp OR asbestosis OR 'fibroses, pulmonary' OR (fibroses, AND pulmonary) OR 'fibrosis, pulmonary' OR (('fibrosis,'/exp OR fibrosis,) AND pulmonary) OR 'pulmonary fibroses' OR (pulmonary AND fibroses) OR 'alveolitis, fibrosing'/exp OR 'alveolitis, fibrosing' OR (('alveolitis,'/exp OR alveolitis,) AND fibrosing) OR 'alveolitides, fibrosing' OR (alveolitides, AND fibrosing) OR 'fibrosing alveolitides' OR (fibrosing AND alveolitides) OR 'fibrosing alveolitis'/exp OR 'fibrosing alveolitis' OR (fibrosing AND ('alveolitis'/exp OR alveolitis)) OR 'idiopathic diffuse interstitial pulmonary fibrosis' OR (idiopathic AND diffuse AND interstitial AND pulmonary AND ('fibrosis'/exp OR fibrosis)) OR 'idiopathic pulmonary fibroses' OR (idiopathic AND pulmonary AND fibroses) OR 'pulmonary fibroses, idiopathic' OR (pulmonary AND fibroses, AND idiopathic) OR 'cryptogenic fibrosing alveolitis'/exp OR 'cryptogenic fibrosing alveolitis' OR (cryptogenic AND fibrosing AND ('alveolitis'/exp OR alveolitis)) OR 'cryptogenic fibrosing alveolitides' OR (cryptogenic AND fibrosing AND alveolitides) OR 'fibrosing alveolitides, cryptogenic' OR (fibrosing AND alveolitides, AND cryptogenic) OR 'pulmonary fibrosis, idiopathic' OR (pulmonary AND ('fibrosis,'/exp OR fibrosis,) AND idiopathic) OR 'fibrosing alveolitis, cryptogenic' OR (fibrosing AND ('alveolitis,'/exp OR alveolitis,) AND cryptogenic) OR 'fibrocystic pulmonary dysplasia' OR (fibrocystic AND pulmonary AND ('dysplasia'/exp OR dysplasia)) OR 'dysplasia, fibrocystic pulmonary' OR (('dysplasia,'/exp OR dysplasia,) AND fibrocystic AND pulmonary) OR 'fibrocystic pulmonary dysplasias' OR (fibrocystic AND pulmonary AND dysplasias) OR 'pulmonary dysplasia, fibrocystic' OR (pulmonary AND ('dysplasia,'/exp OR dysplasia,) AND fibrocystic) OR 'idiopathic fibrosing alveolitis, chronic form' OR (idiopathic AND fibrosing AND ('alveolitis,'/exp OR alveolitis,) AND chronic AND ('form'/exp OR form)) OR 'familial idiopathic pulmonary fibrosis' OR (familial AND idiopathic AND pulmonary AND ('fibrosis'/exp OR fibrosis)) OR 'idiopathic pulmonary fibrosis, familial' OR (idiopathic AND pulmonary AND ('fibrosis,'/exp OR fibrosis,) AND familial) OR 'usual interstitial pneumonia'/exp OR 'usual interstitial pneumonia' OR (usual AND interstitial AND ('pneumonia'/exp OR pneumonia)) OR 'interstitial pneumonia, usual' OR (interstitial AND ('pneumonia,'/exp OR pneumonia,) AND usual) OR 'usual interstitial pneumonias' OR (usual AND interstitial AND pneumonias) OR 'interstitial pneumonitis, usual' OR (interstitial AND ('pneumonitis,'/exp OR pneumonitis,) AND usual) OR 'pneumonitides, usual interstitial' OR (pneumonitides, AND usual AND interstitial) OR 'pneumonitis, usual interstitial' OR (('pneumonitis,'/exp OR pneumonitis,) AND usual AND interstitial) OR 'usual interstitial pneumonitides' OR (usual AND interstitial AND pneumonitides) OR 'usual interstitial pneumonitis' OR (usual AND interstitial AND ('pneumonitis'/exp OR pneumonitis)) OR asbestoses OR 'pulmonary fibrosis - from asbestos exposure' OR (pulmonary AND ('fibrosis'/exp OR fibrosis) AND - AND from AND ('asbestos'/exp OR asbestos) AND ('exposure'/exp OR exposure)) OR 'idiopathic interstitial pneumonitis - from asbestos exposure' OR (idiopathic AND interstitial AND ('pneumonitis'/exp OR pneumonitis) AND - AND from AND ('asbestos'/exp OR asbestos) AND ('exposure'/exp OR exposure))
#2	'quercetin'/exp OR quercetin OR dikvertin
#3	(#1) AND (#2)

Table SIV. Search strategy for Ovid.

Search no.	Ovid
#1	(Pulmonary fibrosis or Idiopathic Pulmonary Fibrosis or Asbestosis or Fibroses, Pulmonary or Fibrosis, Pulmonary or Pulmonary Fibroses or Alveolitis, Fibrosing or Alveolitides, Fibrosing or Fibrosing Alveolitides or Fibrosing Alveolitis or Idiopathic Diffuse Interstitial Pulmonary Fibrosis or Idiopathic Pulmonary Fibroses or Pulmonary Fibroses, Idiopathic or Cryptogenic Fibrosing Alveolitis or Cryptogenic Fibrosing Alveolitides or Fibrosing Alveolitides, Cryptogenic or Pulmonary Fibrosis, Idiopathic or Fibrosing Alveolitis, Cryptogenic or Fibrocystic Pulmonary Dysplasia or Dysplasia, Fibrocystic Pulmonary or Fibrocystic Pulmonary Dysplasias or Pulmonary Dysplasia, Fibrocystic or Idiopathic Fibrosing Alveolitis, Chronic Form or Familial Idiopathic Pulmonary Fibrosis or Idiopathic Pulmonary Fibrosis, Familial or Usual Interstitial Pneumonia or Interstitial Pneumonia, Usual or Usual Interstitial Pneumonias or Interstitial Pneumonitis, Usual or Pneumonitides, Usual Interstitial or Pneumonitis, Usual Interstitial or Usual Interstitial Pneumonitides or Usual Interstitial Pneumonitis or Asbestoses or Pulmonary Fibrosis - from Asbestos Exposure or Idiopathic Interstitial Pneumonitis - from Asbestos Exposure).af.
#2	(Quercetin or 3,3',4',5,7-Pentahydroxyflavone or Dikvertin).af.
#3	(#1) AND (#2)

Table SV. Search strategy for Web of Science.

Search no.	Web of Science
#1	(((((((((((((((((((((((((((((((((((TS=(Pulmonary fibrosis)) OR TS=(Idiopathic Pulmonary Fibrosis)) OR TS=(Asbestosis)) OR TS=(Fibroses, Pulmonary)) OR TS=(Fibrosis, Pulmonary)) OR TS=(Pulmonary Fibroses)) OR TS=(Alveolitis, Fibrosing)) OR TS=(Alveolitides, Fibrosing)) OR TS=(Fibrosing Alveolitides)) OR TS=(Fibrosing Alveolitis)) OR TS=(Idiopathic Diffuse Interstitial Pulmonary Fibrosis)) OR TS=(Idiopathic Pulmonary Fibroses)) OR TS=(Pulmonary Fibroses, Idiopathic)) OR TS=(Cryptogenic Fibrosing Alveolitis)) OR TS=(Cryptogenic Fibrosing Alveolitides)) OR TS=(Fibrosing Alveolitides, Cryptogenic)) OR TS=(Pulmonary Fibrosis, Idiopathic)) OR TS=(Fibrosing Alveolitis, Cryptogenic)) OR TS=(Fibrocystic Pulmonary Dysplasia)) OR TS=(Dysplasia, Fibrocystic Pulmonary)) OR TS=(Fibrocystic Pulmonary Dysplasias)) OR TS=(Pulmonary Dysplasia, Fibrocystic)) OR TS=(Idiopathic Fibrosing Alveolitis, Chronic Form)) OR TS=(Familial Idiopathic Pulmonary Fibrosis)) OR TS=(Idiopathic Pulmonary Fibrosis, Familial)) OR TS=(Usual Interstitial Pneumonia)) OR TS=(Interstitial Pneumonia, Usual)) OR TS=(Usual Interstitial Pneumonias)) OR TS=(Interstitial Pneumonitis, Usual)) OR TS=(Pneumonitides, Usual Interstitial)) OR TS=(Pneumonitis, Usual Interstitial)) OR TS=(Usual Interstitial Pneumonitides)) OR TS=(Usual Interstitial Pneumonitis)) OR TS=(Asbestoses)) OR TS=(Pulmonary Fibrosis - from Asbestos Exposure)) OR TS=(Idiopathic Interstitial Pneumonitis - from Asbestos Exposure) and Preprint Citation Index (Exclude – Database)
#2	((TS=(Quercetin)) OR TS=(3,3',4',5,7-Pentahydroxyflavone)) OR TS=(Dikvertin) and Preprint Citation Index (Exclude – Database)
#3	(#1) AND (#2)
